# Optimising the medical management of hyperglycaemia in type 2 diabetes in the Middle East: pivotal role of metformin

**DOI:** 10.1111/j.1742-1241.2009.02235.x

**Published:** 2009-01

**Authors:** M Al-Maatouq, M Al-Arouj, S H Assaad, S N Assaad, S T Azar, A A K Hassoun, N Jarrah, S Zatari, K G M M Alberti

**Affiliations:** 1 King Khalid University Hospital, King Saud UniversityRiyadh, Saudi Arabia; 2 Dasman Center for Research and Treatment of DiabetesDasman, Kuwait; 3 Alexandria UniversityAlexandria, Egypt; 4 American University of BeirutBeirut, Lebanon; 5 Joslin Diabetes CenterDubai, United Arab Emirates; 6 Mutah UniversityMutah, Karak, Jordan; 7 Al Hada Armed Forces HospitalTaif, Saudi Arabia; 8 Imperial CollegeLondon, UK

## Abstract

**Aims:**

Increases in the prevalence of type 2 diabetes will likely be greater in the Middle East and other developing countries than in most other regions during the coming two decades, placing a heavy burden on regional healthcare resources.

**Methodology:**

Medline search, examination of data from major epidemiological studies in the Middle Eastern countries.

**Results:**

The aetiology and pathophysiology of diabetes appears comparable in Middle Eastern and other populations. Lifestyle intervention is key to the management of diabetes in all type 2 diabetes patients, who should be encouraged strongly to diet and exercise. The options for pharmacologic therapy in the management of diabetes have increased recently, particularly the number of potential antidiabetic combinations. Metformin appears to be used less frequently to initiate antidiabetic therapy in the Middle East than in other countries. Available clinical evidence, supported by current guidelines, strongly favours the initiation of antidiabetic therapy with metformin in Middle Eastern type 2 diabetes patients, where no contraindications exist. This is due to its equivalent or greater efficacy relative to other oral antidiabetic treatments, its proven tolerability and safety profiles, its weight neutrality, the lack of clinically significant hypoglycaemia, the demonstration of cardiovascular protection for metformin relative to diet in the UK Prospective Diabetes Study and in observational studies, and its low cost. Additional treatments should be added to metformin and lifestyle intervention as diabetes progresses, until patients are receiving an intensive insulin regimen with or without additional oral agents.

**Conclusions:**

The current evidence base strongly favours the initiation of antidiabetic therapy with metformin, where no contraindications exist. However, metformin may be under-prescribed in the Middle East.

**What’s known**

- The burden of diabetes is high in the Middle East.
- The Middle East has largely been overlooked by guideline writers.

**What’s new**

- Type 2 diabetes is often sub-optimally managed in the Middle East.
- The pathophysiology of the disease in Middle Eastern patients resembles that in other populations, but few clinical trials recruited Middle Eastern populations.
- Metformin appears to be under-used in the region, and should be considered as initial pharmacologic therapy, in line with international guidelines for the management of type 2 diabetes.

## Introduction

Evidence-based guidelines are necessary to the application of treatments for type 2 diabetes and to ensure best practice in diabetes management (Table 1) (1–6). To date, the only global guideline for the management of type 2 diabetes is that proposed by the International Diabetes Federation (IDF) (1), although other guidelines with regional influence have appeared, including the transatlantic consensus guideline proposed jointly by the American Diabetes Association (ADA) and the European Association for the Study of Diabetes (EASD) (2,3). An expert group in the Middle East adapted the ADA/EASD recommendations for use in the Arab world in 2007, particularly with reference to the low rate of medical insurance coverage and the variable provision of specialist diabetes services and diabetes education across the region (4).

**Table 1:** Overview of leading guidelines for the management of type 2 diabetes

					Recommendations for initiating pharmacologic antidiabetic therapy*	
Reach	Guideline	Year	Goal HbA_1c_ (%)	BMI definition of overweight	Overweight	Non-overweight
Global	IDF (1)	2005	6.5	None given*	Metformin preferred	Metformin or SU
Transatlantic	ADA/EASD (2,3)	2008†	7.0	None given	Metformin preferred‡	
Regional	Middle East (ADA/EASD) (4)	2007	7.0	None given	Metformin preferred‡	
	Asia-Pacific (IDF) (5)	2005	6.5	Ethnic-specific‡	Metformin	Metformin, TZD, SU/meglitinide, AGI
	Latin America (ALAD) (6)	2000	7.0	≥ 27 kg/m^2^	Metformin	SU

* Oral antidiabetic therapy is prescribed after a trial of lifestyle intervention except for American Diabetes Association (ADA) /European Association for the Study of Diabetes (EASD) and Middle-eastern guideline where metformin should be prescribed alongside lifestyle intervention at the time of diagnosis of type 2 diabetes.

† First issued in 2006 and updated in 2008.

‡ The International Diabetes Federation (IDF) now propose ethnic-specific cut-off values for waist circumference to diagnose abdominal obesity. AGI, α-glucosidase inhibitor; ALAD, asociación latinoamericana de diabetes; SU, sulfonylurea; TZD, thiazolidinedione.

**Figure 1:**
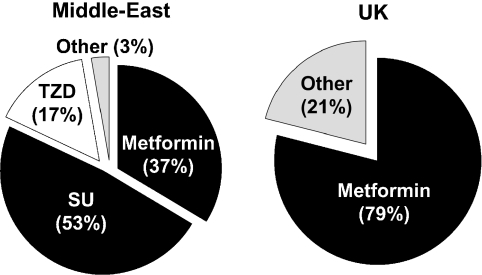
Use of metformin as initial oral antidiabetic pharmacotherapy in the Middle East (2008) and the UK (2007).

These guidelines, described in detail below, have increasingly favoured the initiation of oral antidiabetic pharmacotherapy with metformin. Although it is difficult to define the frequency of use of metformin as initial pharmacotherapy for type 2 diabetes in the Middle East, this treatment may be underused in the region. Survey evidence suggests that about four-fifths of the recently diagnosed type 2 diabetes patients in the UK start oral antidiabetic drug treatment with metformin, compared with only about one-third in the Middle East (Figure 1). It appears, therefore, that metformin may be underused in the Middle East, compared with western countries, and that a consensus guideline on diabetes management directly relevant to the countries of the region may be required. This article considers the nature and management of type 2 diabetes in the region, with particular reference to the therapeutic use of metformin, and proposes recommendations on the initiation of antidiabetic therapy for Middle-Eastern patients with type 2 diabetes.

### Pathophysiology of type 2 diabetes

The progression of hyperglycaemia in type 2 diabetes is driven by β-cell dysfunction occurring against a background of insulin resistance (1,2). The development of insulin resistance is usually an early event in the pathogenesis of type 2 diabetes and requires increased secretion of insulin to maintain euglycaemia. As β-cell function continues to decline, insufficient insulin is secreted to control blood glucose adequately and chronic fasting and/or postprandial hyperglycaemia becomes established. Eventually, insulin secretion often declines to the point where exogenous injections of insulin are required. Data from the UK Prospective Diabetes Study (UKPDS) suggest that about half of a patient’s original β-cell function have already been lost by the time diabetes is diagnosed (7).

Most oral antidiabetic treatments target insulin resistance or β-cell dysfunction as their primary mechanisms of action (2). Metformin addresses insulin resistance primarily in the liver and skeletal muscle and mainly reverses hyperglycaemia through a reduction in hepatic glucose production, while the thiazolidinediones increase whole-body insulin-mediated glucose disposal to a greater extent than metformin (8). Sulfonylureas and drugs acting via the incretin system, dipeptidyl peptidase-4 (DPP-4) inhibitors (incretin enhancers) and glucagon-like peptide 1 (GLP-1) agonists (incretin mimetics), increase insulin secretion with the incretin drugs also normalising glucagon secretion. Finally, α-glucosidase inhibitors slow and smooth the absorption of glucose from the gastrointestinal tract, reducing postprandial hyperglycaemia. It is important to note that hyperglycaemia *per se* is toxic to the β-cell; thus, any treatment which reduces the severity of hyperglycaemia is likely to improve β-cell function to some extent over the short term.

### Type 2 diabetes in the Middle East

#### Increasing burden of diabetes

**Figure 2:**
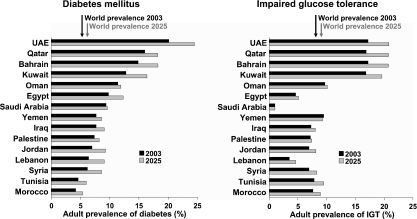
Increasing burden of dysglycaemia in the Middle East. Data shown are from the 15 countries from the International Diabetes Federation (IDF) Middle East and North Africa region with the highest adult (20–79 years) prevalence of diabetes in 2003, according to the IDF E-atlas of Diabetes (9). Data from Armenia and Pakistan were omitted for clarity. IGT: impaired glucose tolerance

The burden of diabetes in the Middle East is high. Figure 2 compares the recently reported prevalence of diabetes in adults (20–79 years) in countries in the region alongside the projected prevalence for 2025, according to the IDF (9). The prevalence of diabetes in most Middle-Eastern countries is already well above average for the world as a whole and is set to increase markedly in the region by 2025. While the worldwide prevalence of diabetes in 2025 will be almost 25% higher than the value in 2003, the prevalence of diabetes in the IDF Middle East and North Africa region is set to increase by 81% during this period. Taking into account the projected increases in populations of these countries, this means that the number of people with diabetes in the Middle East is set to be more than double (9).

When considering the data shown in Figure 2, it is important to note that the results of epidemiological surveys depend critically on the precise methodology used and the nature of the populations studied. Moreover, the projected prevalence estimates for 2025 are based on projected changes in population and body weight. These estimates should be treated as a guide rather than as a definitive and quantitative determinant of prevalence and should be considered alongside other studies, wherever available. For example, the age-adjusted prevalence of diabetes among a nationally representative population of 17,232 subjects in Saudi Arabia was 22% (10), which is higher than the estimate from the IDF shown in Figure 2. Another study based on a nationally representative sample of 5844 subjects in the United Arab Emirates in 1999–2000 demonstrated an age-standardised prevalence of diabetes of 21% (22% in men and 21% in women), which is closer to the current estimate from the IDF (11). A high and relatively transient expatriate population also complicates measurements of disease prevalence in the Middle East. In the study described above, the prevalence of diabetes was higher in Emirati citizens than in expatriates (25% vs. 13–19%).

A substantial burden of non-diabetic dysglycaemia [impaired glucose tolerance (IGT) or impaired fasting glucose (IFG)] provides a large reservoir of patients at high risk of developing type 2 diabetes in the Middle East, as elsewhere (Figure 2) (9). A high prevalence of undiagnosed diabetes also contributes to the problem. Population-based surveys in Saudi Arabia, Egypt and the United Arab Emirates have demonstrated a high prevalence of these conditions, in addition to previously diagnosed diabetes (10,12,13). Urbanisation, followed by access to high-energy foods and adoption of sedentary habits, is an important underlying cause of the increasing prevalence of diabetes and associated cardiovascular disease in the Middle East, as in other regions (10,14). The quality of diet is variable; however, a study in randomly selected non-diabetic Egyptian subjects concluded that their diet was similar to that recommended by the Diabetes and Nutrition Study Group of the European Association for the Study of Diabetes (EASD) (15).

The cost of managing diabetes is high. The majority of the cost of managing diabetes arises from the management of diabetic complications, especially in hospital. In the main analysis of the UKPDS, treatment of complications accounted for 63% of overall within-trial costs in the group randomised to receive conventional (diet treatment) (16). Similar data were calculated for overweight patients, where the management of complications accounted for 74% of overall within-trial costs in the diet group (17). Few health economic analyses have been conducted in the Middle East. Patients with diabetes account for 2.6% of all hospital admissions and 3.5% of all hospital stays in Saudi Arabia (18). A report from a health insurance company in Abu Dhabi, reported in the press, claimed recently that the cost of managing diabetes in the UAE will reach Dh 10 billion (US $2.7 billion) by 2020 (19). There is no doubt that the economic burden of diabetes will continue to increase as the prevalence of the disease increase in Middle-Eastern countries.

#### Clinical characteristics of diabetes in the Middle East

The clinical characteristics of diabetes have not been well studied in many countries of the region. However, it is clear that obesity (especially abdominal obesity), family history of diabetes and other commonly occurring cardiometabolic risk factors are associated with a significant increase in the risk of developing type 2 diabetes in this population, as has been demonstrated elsewhere (20,21). A large survey in Saudi Arabia found that 36% of subjects (26% of men and 44% of women) were obese (22). However, a genetic predisposition to type 2 diabetes, exacerbated by a tendency towards consanguinity and a relatively high prevalence of cardiometabolic risk factors associated with the metabolic syndrome, may contribute to the high prevalence of diabetes in the Middle East (10,23).

Indeed, cardiometabolic risk factors are common among Middle-Eastern type 2 diabetes patients (24–27). Large surveys in Saudi Arabia (*n*= 17,230, age 30–70 years) (25) and Egypt (*n*= 7915, age > 25 years) demonstrated a prevalence of hypertension of 26%, in each case (only 58% had normal blood pressure according to JNC-V guidelines in Egypt, with the remainder having high-normal blood pressure) (26,27). Only 38% of patients with hypertension were aware of their condition in Egypt (27). Hypertension was strongly associated with obesity and other cardiometabolic risk factors in both countries. The atherogenic dyslipidaemia phenotype is also common, and about one patient in five among a randomly selected Saudi cohort of 507 patients with type 2 diabetes was found to have low HDL-C [defined as < 35 mg/dl (0.9 mmol/l) in men and < 45 mg/dl (1.2 mmol/l) in women] (28). Similarly, the prevalence of HDL-C < 35 mg/dl (0.9 mmol/l) was 25% in 1733 men and women aged > 25 years surveyed in the Egyptian National Hypertension Project (29).

Epidemiological studies in the region may be complicated by the relatively high proportion of expatriate workers in some Middle-Eastern countries. However, another study from Saudi Arabia showed that the prevalence of individual cardiometabolic risk factors (dyslipidaemia, smoking, obesity, high blood pressure and poor glycaemic control) differed little among Saudi and non-Saudi patients with type 2 diabetes (30).

Survey evidence suggests a low success rate in achieving targets for cardiometabolic risk factor control, with low proportions of a Saudi population attending a hospital clinic achieving blood pressure < 130/85 mmHg (60%), triglycerides < 2.3 mmol/l (200 mg/dl) (35%), body mass index < 27 kg/m^2^ (46%), LDL-C < 2.6 mmol/l (100 mg/dl) (18%), or HDL-C > 1.1 mmol/l (43 mg/dl) (33%) (31). In the Egyptian National Hypertension Project, only 24% of patients received antihypertensive medication, and only 8% of patients achieved JNC-V goals for blood pressure (27). A similar low rate of control of hypertension was observed in type 2 diabetes patients in Jordan (32).

Although the association between type 2 diabetes and cardiovascular risk has not been studied as intensively in the Middle East as in other populations, there is evidence that a comparable relationship exists between a diagnosis of diabetes and adverse cardiovascular outcomes. The prevalence of coronary artery disease in Emirati subjects tended to increase consistent with the severity of dysglycaemia, from prediabetes (4.7%) to undiagnosed (5.0%) or diagnosed (10.5%) diabetes (12). A similar trend was observed for peripheral vascular disease (3.6%, 5.0% and 11.1% respectively) (12). Type 2 diabetes is also a powerful risk factor for ischaemic stroke in the region (33). Elevated fasting blood glucose has been shown to promote the progression of coronary artery disease in Lebanese patients (34).

**Figure 3:**
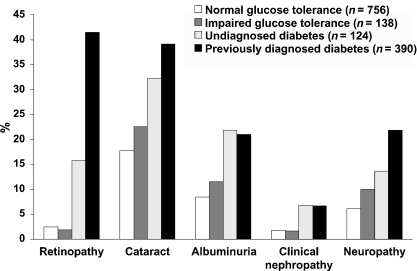
Prevalence of microvascular complications or cataract in a survey in Egypt. Albuminuria was defined as a urinary albumin:creatinine ratio > 100 mg/g. Clinical nephropathy was defined as urinary albumin:creatinine ratio > 300 mg/g. Drawn from data presented by Herman et al. (13)

The association between long-term hyperglycaemia and an increased risk of microvascular diabetic complications appears comparable for the Middle East and elsewhere. A survey in Egypt confirmed the high prevalence of microvascular complications in an Arab population with diabetes (Figure 3) (13). Elsewhere, a survey in 1952 Saudi type 2 diabetes patients admitted to hospital between 1989 and 2004 found an incidence of retinopathy of 32% (35), with a corresponding figure of 24% from a survey in Kuwait (2006) (36). These figures appear somewhat lower than the prevalence of retinopathy in the newly diagnosed population of the UKPDS, where the prevalence of retinopathy was 36% at baseline (37), although they were considerably higher than the corresponding figure of 8% for the Fenofibrate Intervention and Event Lowering in Diabetes (FIELD) Study (38). It is important to note that different definitions of retinopathy may make direct comparisons difficult between individual trials. Nevertheless, the risk of progression of retinopathy was increased by an increasing duration of diabetes, sub-optimal glycaemic control and higher levels of blood pressure in all these analyses in an apparently similar manner. A similar relationship holds for nephropathy: although the incidence of diabetes-related end-stage renal failure appears to be lower in the Middle East than in western countries, under-reporting of the true prevalence may have underestimated the true burden of this condition (39). A population-based survey in the United Arab Emirates demonstrated a prevalence of neuropathy of 35% among patients with diabetes (12), while a single-centre survey at a Saudi Arabian diabetes clinic found that 56% of diabetic patients had symptomatic neuropathy, with about half of the remainder having subclinical, asymptomatic neuropathy (40). Figure 3 shows the prevalence of neuropathy in Egypt in patients stratified for different severities of dysglycaemia (13).

#### Poor glycaemic control is common in the Middle East

Poor glycaemic control is common in the region. The study conducted in Egypt, described above, found mean HbA_1C_ values of 9.2% in patients with previously diagnosed diabetes and 8.7% in patients with previously undiagnosed diabetes (13). A retrospective survey of 991 Saudi type 1 or type 2 diabetes patients attending hospital diabetes clinics revealed ‘excellent’ blood glucose control [4–7 mmol/l (72–126 mg/dl)] in only 21%, with ‘poor’ control [> 10 mmol/l (180 mg/dl)] in > 40% (41). Another survey from the same country showed that only 27% of type 2 diabetes patients achieved HbA_1C_ < 7.0% (31). Somewhat better glycaemic control was demonstrated in patients with diagnosed (mean HbA_1C_ 8.3%) or undiagnosed (mean HbA_1C_ 6.7%) diabetes in the United Arab Emirates (12).

Important barriers to achieve successful treatment outcomes differ between the Middle East and Europe. Middle-Eastern states are far from homogenous in demographic terms, with highly developed and westernised areas often coexisting with relatively undeveloped regions with high rates of poverty (14). Access to healthcare, along with other basic amenities, is often restricted, particularly in remote rural areas, and the prevalence of cardiometabolic risk factors may differ markedly between regions of the same country (42). Other socioeconomic factors, such as cultural traditions, education and income are also important determinants of health outcomes. Thus, the level of education was a powerful predictor of knowledge of the both causes of coronary heart disease and of strategies to improve cardiovascular health in a population of Saudi patients attending a primary care health centre, although less than half of the population as a whole knew about such issues (43).

## Optimising the management of type 2 diabetes: metformin in comparison with other oral antidiabetic therapies

### Efficacy and tolerability

Table 2 shows an overall comparison of key properties of metformin that are relevant to its overall risk: benefit ratio, in comparison with other classes of oral antidiabetic therapy (44). Metformin is as effective as other oral antidiabetic agents, with little or no potential for clinically significant hypoglycaemia, in contrast to sulfonylureas or meglitinides, which are associated with a relatively high incidence of hypoglycaemia (44). Metformin can be combined with members of any class of oral antidiabetic agents, including insulin or the new incretin enhancers and incretin mimetics. Indeed, metformin itself potentiates the actions of endogenous GLP-1 to a clinically significant extent, through either inhibition of DPP-4 (45) or enhancement of GLP-1 secretion (46).

**Table 2:** Comparison of classes of oral antidiabetic agents (45)

	Met	SU	Meg	TZD	DPP-4 inh	GLP-1 agonists	AGI
Expected ↓HbA_1c_*	1.0–2.0	1.0–2.0	0.5–1.5	0.5–1.4	0.5–0.8	0.5–1.0	0.5–0.8
Hypoglycaemia risk	Very low	High	High	Very low	Low	Low	Very low
Effects on body weight	Neutral or weight loss	Weight gain	Weight gain	Weight gain	Neutral	Weight loss	Neutral
Other side effects	GI symptoms	None	None	Oedema	None	Nausea	Frequent GI symptoms
Other safety issues	Lactic acidosis	None	None	Heart failure, fractures	Skin, immune disorders?	Pancreatitis?	None
CV outcomes	↓CV events (UKPDS)	Neutral	Neutral	Conflicting data	No data	No data	↓CV events (meta analysis)
Cost	Low (generic)	Low (generic)	Low (generic)	Very high	Very high	Very high	High

Met, metformin; SU, sulfonylurea; Meg, meglitinide; TZD, thiazolidinediones; AGI, α-glucosidase inhibitors; inh, inhibitors.

* As proposed in the joint guideline proposed by the American Diabetes Association and the European Association for the Study of Diabetes (2) See text for other references.

Importantly, metformin is not associated with increases in body weight, unlike sulfonylureas and thiazolidinediones; indeed, body weight is often reduced during metformin treatment (47,48). The main side effects of metformin occur in the gastrointestinal system (particularly diarrhoea). These can be minimised by initiating treatment at a low dose (500 mg) and titrating cautiously [the maximum daily dosage for most patients will be in the region of 1700 mg (2 × 850 mg) or 2000 mg (4 × 500 mg or 2 × 1000 mg)]. An extended-release formulation has been shown to improve gastrointestinal tolerability in patients unable to tolerate standard immediate-release metformin (49). Incretin mimetics also induce gastrointestinal side effects (mainly nausea), although this is described as transient.

### Safety

Many publications have linked metformin with an increased risk of lactic acidosis (44). A Cochrane review confirmed the similar risk of lactic acidosis for metformin and non-metformin treatments for diabetes, with upper estimates of the risk of 8.4 and 9 patients per 100,000 patient-years respectively (50). A more recent meta analysis, including three additional randomised, controlled studies, has confirmed this outcome (48). Finally, the Comparative Outcomes Study of Metformin Intervention vs. Conventional (COSMIC) Approach Study, a 1-year randomised comparison of metformin and other diabetes treatments conducted under usual care conditions in 8732 patients, found no cases of lactic acidosis (51). Accordingly, the risk of lactic acidosis with metformin is not higher than that with other antidiabetic therapies, when the contraindications and precautions of metformin are respected.

The potential of thiazolidinediones to increase cardiovascular risk (particularly myocardial infarction) is controversial, and previous meta analyses have provided conflicting results, especially with respect to rosiglitazone (52–54). Pioglitazone has not been associated with increased cardiovascular risk to the same extent and has been shown to exert a modest improvement in secondary cardiovascular end-points in a randomised trial (see below). Current ADA/EASD management guidelines in type 2 diabetes suggest that rosiglitazone should be avoided and states that the use of thiazolidinediones in general is less well-validated than the addition of a sulfonylurea or insulin to metformin when antidiabetic combination therapy is required (2). Thiazolidinediones are also associated with an increased risk of oedema-associated heart failure (54,55) and have also been shown to slightly but significantly increase the risk of distal limb fractures in women (55). These effects have been demonstrated with both rosiglitazone and pioglitazone.

Incretin enhancers act via blockade of DPP-4, which is present in immune cells. Although there is currently no hard clinical evidence to support an association between DPP-4 inhibition and adverse effects on the immune system, a Cochrane analysis has called for more data (56), and the ADA/EASD guidelines acknowledge the theoretical potential for immune dysregulation with these agents (2,3). Preclinical studies suggest that a high selectivity for DPP-4 over other similar enzymes is necessary to reduce the risk of cutaneous adverse events, and the US regulatory authorities are currently reviewing the therapeutic profile of vildagliptin with regard to this issue (57). Cases of pancreatitis have been reported with incretin mimetics, although the clinical significance of these observations is uncertain.

### Cardiovascular outcomes

**Figure 4:**
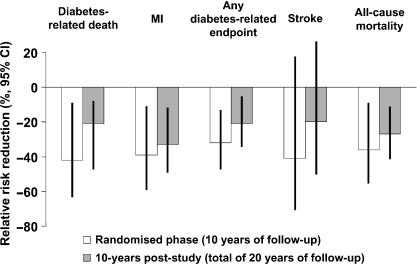
Effects of metformin on clinical cardiovascular outcomes in the UKPDS. The reference group for risk reductions was patients randomised to diet-based treatment. All risk reductions were significantly different except those for stroke. MI: myocardial infarction. Drawn from data presented in Refs (59,60)

Randomisation to metformin in the UKPDS was associated with significant improvement in a range of cardiovascular end-points, with these benefits maintained after 10 years of posttrial monitoring during which patients returned to the usual care of their physician (Figure 4) (58,59). A recent randomised, placebo-controlled trial evaluated the effects of metformin on clinical outcomes in 390 insulin-treated type 2 diabetes patients followed for 4.3 years (60). Metformin did not significantly influence the primary cardiovascular end-point (a composite of microvascular and macrovascular end-points), but significantly reduced the risk of a secondary end-point comprising a composite of macrovascular end-points by 40% [hazard ratio 0.60 (95% CI, 0.40–0.92), p = 0.04].

Additional observational analyses, reviewed elsewhere (44), have added to the evidence base for cardiovascular protection with metformin. One of these was a *post hoc* analysis of the prevention of restenosis with tranilast and its outcomes (PRESTO) trial, involving analysis of data from 1997 patients with type 2 diabetes at baseline who received either metformin or other oral antidiabetic treatments that do not influence the action of insulin as their primary mechanism (i.e. patients receiving a thiazolidinedione were excluded) (61). Patients in the metformin group benefited from lower adjusted risks of any clinical event [risk reduction (RR) 28%, p =0.005], myocardial infarction (RR 69%, p =0.002) or all-cause mortality (RR 61%, p =0.007).

Pioglitazone improved secondary cardiovascular end-points in the PROACTIVE trial (62–64), although the continuing controversy regarding the cardiovascular safety of these agents is discussed above. Although a meta analysis of acarbose trials suggested a reduction in cardiovascular events (65), no other class of oral antidiabetic agent has demonstrated unequivocal cardiovascular protection. The long-term cardiovascular safety profile of agents acting via the incretin system is largely unknown, because of the limited clinical experience available for these agents.

### Cost

The cost of treatment is another important issue influencing the access of many patients to healthcare. Metformin and some sulfonylureas are available as generic preparations; therefore limiting their cost. However, the quality of some of these generic preparations remains a cause for concern. Thiazolidinediones, α-glucosidase inhibitors and agents acting via the incretin system are branded preparations and are thus more expensive.

### Additional properties of metformin

The principal factors in selecting an oral antidiabetic agent are its antihyperglycaemic efficacy, together with other properties that suggest a potential long-term benefit (2,3). Clinical studies have documented beneficial metabolic effects potentially in metformin-treated patients in a range of conditions associated with insulin resistance (44). These include non-alcoholic fatty liver disease (and its more prognostically serious form, non-alcoholic steatohepatitis, which are closely associated with the metabolic syndrome) (66), polycystic ovary syndrome [(PCOS) the most common cause of infertility in western populations] (67), HIV-associated lipodystrophy (secondary to an induced form of insulin resistance caused by protease inhibitors) (68) and the prevention of weight gain induced by second-generation antipsychotic agents (69). Observational evidence also supports an antineoplastic effect of metformin, consistent with its activation by metformin of the enzyme, AMP-activated protein kinase, which in turn activates a tumour suppressor, LKB1 (70,71). To date, metformin is not indicated for these conditions, although management guidelines in this area identify a place for metformin in the management of PCOS (44). Although these effects are speculative at this time, they may be of relevance to individual patients displaying these conditions at presentation, and further clinical research will properly define the potential of metformin in these areas.

The role of oral antidiabetic therapy in the management of gestational diabetes requires further research, not least as women with PCOS may become pregnant while receiving treatment for this condition. At present, insulin is the mainstay of the management of gestational diabetes, and oral agents are discontinued. However, a recent systematic review has shown that the use of oral antidiabetic therapy, including metformin, was not associated with an increased risk of adverse foetal outcomes, and a lower rate of neonatal hypoglycaemia, relative to insulin (72).

### Guidelines

Data on the effectiveness of antidiabetic interventions in Middle-Eastern subjects are scarce. Accordingly, it is reasonable to assess individual therapies on the basis of their therapeutic profiles demonstrated in other populations, especially as the aetiology and pathogenesis of diabetes in the Middle East and other regions appear comparable (as described above). Table 1 summarises the key features of several leading guidelines. A joint guideline issued by the American Diabetes Association (ADA) and the EASD (2,3), and an adaptation of these guidelines for Middle-Eastern patients (4), recommend immediate prescription of metformin alongside lifestyle intervention (this therapy is continued throughout the course of diabetes) for all patients with type 2 diabetes and HbA_1C_ above 7.0% who are able to receive metformin. This is followed, when required, by combination therapy with a sulfonylurea or basal insulin [i.e. neutral protamine hagedorn (NPH) insulin or newer insulin analogues], which are considered to be well-validated therapies. Where metformin-sulfonylurea combinations do not control glycaemia sufficiently, patients should receive metformin plus basal insulin. Combinations based on metformin with pioglitazone (rosiglitazone is not recommended following recent concerns over the risk:benefit profile of this agent) or an incretin mimetic (GLP-1 agonist) are included as ‘less well-validated therapy’. Eventually, all patients requiring insulin should progress to treatment with lifestyle intervention, metformin and an intensive insulin regimen.

The major international guideline issued by the IDF (1) and an adaptation of these guidelines for the Asian-Pacific region (5) recommended prescription of metformin as first-line pharmacological antidiabetic therapy for patients without contraindications, who did not respond sufficiently well to a trial of lifestyle intervention. Sequential intensification of oral antidiabetic therapy is required up to and including insulin-based treatment. Importantly, the lifestyle intervention is maintained throughout the course of the disease, as in the ADA/EASD guideline. The IDF published a guideline specifically addressing the control of postmeal glucose in 2007 (73). This guideline recognises the importance of postprandial glucose as a risk factor for long-term diabetic complications and advocates the control of 2-h postmeal glucose to <7.8 mmol/l (140 mg/dl).

Ramadan is a religious observance of the Muslim faith that involves abstinence from food and drink between dawn and sunset for a period of one month. Fasting during Ramadan alters the delivery of antidiabetic treatment for an estimated 40–50 million Muslim patients with diabetes worldwide (74). Guidance on optimising blood glucose control, while minimising the risk of hypoglycaemia, hyperglycaemia, ketoacidosis, dehydration and thrombosis, is required for these patients (74,75). It is important that patients should not fast if they are unwell and should end their fast immediately if blood glucose falls below 60 mg/dl (3.3 mmol/l). The fast should also be broken if blood glucose is < 70 mg/dl (3.9 mmol/l), especially if the patient is taking insulin or insulin secretagogues. Oral antidiabetic agents with a low risk of hypoglycaemia, such as metformin, are preferred. Individually tailored insulin regimens, with divided doses or basal and/or short- or rapid-acting insulins before the predawn and postsunset meals, may reduce the risk of hypoglycaemia for patients who require treatment with insulin. Educational counselling and maintaining contact with the physician during Ramadan are also important.

### Recommendations for type 2 diabetes management in the Middle East

**Figure 5:**
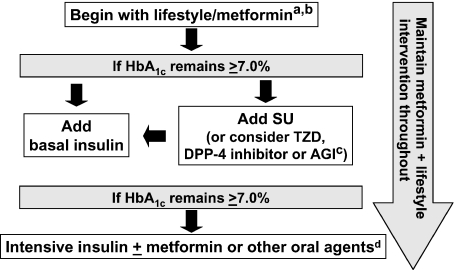
Proposed algorithm for the management of type 2 diabetes in the Middle East. ^a^Insulin may be required initially to stabilise patients presenting with severe hyperglycaemia. ^b^A short trial of lifestyle intervention (e.g. 1 month) may be given before starting metformin (lifestyle intervention and metformin may be co-prescribed where additional clinic visits are problematic for the patient). ^c^When appropriate for an individual patient. ^d^Avoid combination of insulin with a thiazolidinedione (TZD) because of increased risk of oedema. Referral to a specialist will be appropriate for some patients

Recommendations for the use of oral antidiabetic therapy in the management of type 2 diabetes are summarised in Figure 5. The initiation of oral antidiabetic pharmacotherapy with metformin, alongside lifestyle intervention, remains the therapeutic strategy best supported by current clinical evidence. This is particularly the case in countries like those of the Middle East, where the cost of medication is an important issue for many patients.

Treatment may be initiated with lifestyle intervention. Even a brief trial of lifestyle intervention, of as little as 1 month, is often useful in indicating the potential of this treatment to impact substantially on hyperglycaemia. A separate trial of lifestyle intervention also emphasises the importance of this treatment in its own right and helps to educate patients that it is not an optional extra to pharmacotherapy. It should be noted; however, that access to healthcare may represent an important barrier to treatment for many Middle-Eastern patients, and that the additional clinic visits necessary to administer a trial of lifestyle intervention may be problematic in some cases. For these patients, metformin may be co-prescribed at this time, as per the ADA/EASD guideline, according to the physician’s judgement. Basal insulin (NPH or analogue) or a sulfonylurea is the preferred next step in treatment intensification, although other agents may be more appropriate for an individual patient (e.g. a patient susceptible to or concerned about hypoglycaemia, with contraindications to or intolerance of metformin). Patients maintained on oral combinations then receive basal insulin, with intensive insulin regimens (with or without oral agents) as the final step.

## Conclusions

The application of evidence-based therapy for patients with type 2 diabetes is essential given the projected large increases in the prevalence of type 2 diabetes in countries of the Middle East. Although the number of treatments for type 2 diabetes has increased in recent years, metformin plus lifestyle intervention remains the preferred strategy for the initiation of oral antidiabetic pharmacotherapy.
